# Portable polygraphic device (Somnocheck micro CARDIO^®^) provides accurate diagnostic information in psychiatric patients at risk for obstructive sleep apnoea: an observational cohort study

**DOI:** 10.1186/s12888-024-06049-8

**Published:** 2024-09-10

**Authors:** Maximilian Bailer, Eva M. Stein, Maximilian I. Sprügel, Stefan Mestermann, Philipp Spitzer, Janine Utz, Sabine Zirlik, Florian S. Fuchs, Johannes Kornhuber

**Affiliations:** 1 Department of Psychiatry and Psychotherapy, Friedrich-Alexander-Universität Erlangen-Nürnberg, Schwabachanlage 6, 91054 Erlangen, Germany; 2 Department of Neurology, Friedrich-Alexander-Universität Erlangen-Nürnberg, Erlangen, Germany; 3 Department of Internal Medicine 1, Friedrich-Alexander-Universität Erlangen-Nürnberg, Erlangen, Germany

**Keywords:** Sleep apnoea, obstructive (OSA), Mental disorders, Portable cardiorespiratory polygraphy, Polysomnography (PSG), Sleep, Mental health

## Abstract

**Background:**

Symptoms of obstructive sleep apnoea (OSA) overlap significantly with those of psychiatric disorders, making accurate diagnosis of OSA challenging within psychiatric settings. Diagnosing OSA in psychiatric patients is crucial because untreated OSA can exacerbate psychiatric symptoms, reduce treatment efficacy, and impair overall quality of life. This study aimed to determine the diagnostic accuracy of a readily accessible procedure for psychiatric patients in a real-world clinical setting by comparing the Somnocheck micro CARDIO^®^ (SCm) portable cardiorespiratory polygraphy device with the gold standard polysomnography (PSG).

**Methods:**

This observational cohort study included consecutive psychiatric patients at intermediate to high risk for OSA based on screening with the STOP-Bang questionnaire, admitted to a single tertiary care centre between June 1, 2016 and December 31, 2022. The Apnoea-Hypopnoea-Index (AHI), Apnoea-Index (AI), Oxygen-Desaturation-Index (ODI), and minimum oxygen saturation were measured sequentially by SCm and PSG.

**Results:**

A total of 57 patients were analysed (median age 62.0 [Interquartile Range (IQR), 51.5–72.5] years; 34 [59.6%] men). Regarding AHI, no significant differences (AHI measured by PSG, median, 16.6 [IQR, 6.2–26.7] vs. AHI measured by SCm, median, 14.9 [IQR, 10.0-22.8]; *p* = 0.812; *r* = 0.71) were found between SCm and PSG. AI, ODI and minimum oxygen saturation differed significantly between SCm and PSG. Using optimised cut-off values (any OSA: AHI_SCm_ ≥ 9.25), SCm showed high sensitivity (0.894) and high specificity (0.800) for the diagnosis of OSA, with an area under the receiver operating characteristic curve of 0.877.

**Conclusions:**

This study found that the SCm portable device was accurate in identifying psychiatric patients with OSA. AHI measurement by SCm provided reliable diagnostic performance in comparison with the gold standard polysomnography. These findings support the integration of polygraphic measurements into the routine sleep assessment of psychiatric patients. Early and accurate diagnosis of OSA in this population can significantly improve the management of both sleep disorders and psychiatric conditions, potentially enhancing overall treatment outcomes and quality of life for these patients.

**Supplementary Information:**

The online version contains supplementary material available at 10.1186/s12888-024-06049-8.

## Background

Obstructive sleep apnoea (OSA) is a very common condition, affecting approximately 9–38% of the general adult population [1] in Western societies, where prevalence rates are increasing due to factors such as obesity and aging [2, 3]. Studies indicate that OSA is even more prevalent in psychiatric patients [4, 5]. For instance, the median prevalence rate of OSA in a cohort of patients with major depressive disorder is reported to be 48.1% [6]. If left untreated, OSA significantly increases mortality [7], impairs quality of life [8] and increases the risk of depression [9]. In psychiatric patients, treatment of OSA with continuous positive airway pressure (CPAP) can help alleviate depressive symptoms and improve mood [10]. Patients with major depressive disorder and comorbid OSA may also respond less well to treatment with antidepressants, underscoring the critical need for accurate diagnosis and tailored treatment [11].

Identifying patients with OSA is therefore particularly important in a psychiatric setting, as it can significantly impact treatment outcomes and overall mental health. However, diagnosing OSA in psychiatric patients is complicated by several factors. Clinical symptoms of OSA such as daytime sleepiness, impaired sleep quality, and alterations in cognition, mood, and general performance [12, 13] overlap with symptoms of psychiatric disorders [14]. These overlapping symptoms can lead to underdiagnosis or misdiagnosis, as they can be falsely attributed solely to the psychiatric condition. While screening tools such as the STOP-Bang questionnaire are often used to identify patients at risk for OSA, thus increasing the likelihood of a positive PSG result [15, 16], limited studies assess the diagnostic validity of screening questionnaires in psychiatric patients, with most validation studies conducted in general or sleep clinic populations [17].

The gold standard to confirm the diagnosis of OSA is overnight polysomnography [18, 19]. However, this method can be relatively expensive and may not always be available. Portable polygraphy devices offer a more accessible and cost-effective alternative to in-laboratory PSG for diagnosing OSA, particularly in patients with a high pre-test probability of moderate to severe OSA or when PSG is not feasible [19, 20].

Despite the availability and general diagnostic accuracy of portable devices [21–24], there is a significant gap in research validating these tools specifically for psychiatric patients: Most portable polygraphy devices are validated for the general population, but lack specific validation for psychiatric patients, especially in real-world clinical settings [23, 25]. Consequently, current sleep medicine guidelines caution against relying solely on portable devices for diagnosing OSA in patients with psychiatric comorbidities [26]. Furthermore, adherence problems are common in psychiatric patients [27]. Easily accessible diagnostic procedures for OSA are therefore particularly important for this population, with ambulatory polygraphy devices offering several advantages, including ease of use, reduced cost, and the convenience of being administered outside traditional clinical settings [28].

This study aims to address these gaps by evaluating the diagnostic accuracy of a portable cardiorespiratory polygraphy device, the Somnocheck micro CARDIO^®^ (SCm) system, compared to the gold standard sleep laboratory PSG among psychiatric patients in a real-world clinical setting.

## Methods

### Study subjects and study design

Consecutive patients with psychiatric disorders admitted to a psychiatric ward of the Psychiatric Clinic, University Hospital Erlangen, Germany, between June 1, 2016 and December 31, 2022, were included in an observational cohort study, the Universitätsklinikum Erlangen study of obstructive sleep apnoea in psychiatric patients (UKER-OSA-PS), to investigate the prevalence and risk factors for obstructive sleep apnoea and to evaluate the validity of a diagnostic algorithm. The study period was chosen because the hospital’s patient records were fully digitalised by June 2016, allowing for comprehensive and accurate data collection from this point onward. To prevent any harm to patients, standard clinical procedures were used throughout, with all diagnostic assessments deemed beneficial for patient care.

### Diagnostic algorithm

Eligible patients were screened ad admission for OSA using the **s**noring, **t**iredness, **o**bserved apnoea, high blood **p**ressure, **B**ody-Mass-Index (BMI), **a**ge, **n**eck circumference, and male **g**ender (STOP-Bang) questionnaire. The STOP section of the questionnaire assesses self-reported symptoms and risk factors, while the Bang section further includes BMI (> 35 kg/m²), age (> 50 years), neck circumference (> 41 cm in females and > 43 cm in males), and male gender. High risk for OSA is defined as positive answers to ≥ 5 questions, intermediate risk as positive answers to 3–4 questions, and low risk as ≤ 2 affirmative answers [15, 16]. Patients with intermediate to high risk for OSA based on screening with the STOP-Bang questionnaire, or if OSA was clinically suspected for other reasons, were investigated with the SCm portable cardiorespiratory polygraphy device. Patients were classified into risk groups based on their SCm testing. According to the manufacturer’s protocol, an AHI of ≥ 10 as determined by the SCm device indicates a moderate to high risk of OSA, warranting further evaluation with by PSG. Patients with a moderate to high risk of OSA on the SCm testing (AHI_SCm_ ≥ 10), or if OSA was clinically suspected for other reasons, were referred to the Sleep Medicine Centre of the Department of Internal Medicine 1, Friedrich-Alexander-Universität Erlangen-Nürnberg, Erlangen, for further diagnostic evaluation including overnight polysomnography.

### Eligibility criteria

Consecutive patients at intermediate to high risk for OSA based on screening with the STOP-Bang questionnaire were included. This study included only patients who consented to the diagnostic procedures and underwent the full diagnostic algorithm. Patients were excluded if SCm cardiorespiratory polygraphy or overnight polysomnography were not available. Patients were excluded if OSA was already diagnosed at admission or if continuous positive airway pressure (CPAP) treatment was initiated between the two measurements. Patients were excluded if the quality of the PSG measurement was insufficient (less than 200 min sleep duration or poor quality noted in medical records).

### Procedures

#### Somnocheck micro CARDIO® portable cardiorespiratory polygraphy device

The Somnocheck micro CARDIO^®^ system (Weinmann GmbH, Hamburg, Germany) portable cardiorespiratory polygraphy device was selected for its ease of use and diagnostic capabilities which allowed the implementation of the diagnostic procedure in a psychiatric setting. The Somnocheck micro CARDIO^®^ system consists of a base unit, a nasal cannula for airflow and snoring measurement, and a pulse oximetry sensor that also enables pulse wave analysis. Using the SCOPER categorization for polygraphy devices, the Somnocheck micro CARDIO^®^ system can be classified as S_0_ (sleep: no direct measurement); C_4_ (cardiovascular: derived from pulse oximetry); O_1x_ (oximetry: finger); P_2_ (position: non-visual measurement); E_4_ (effort: other effort measurement: derived from pulse wave analysis); R_2_ (respiratory: nasal pressure) [22, 29]. The data from the SCm cardiorespiratory polygraphy was analysed using the software provided by the manufacturer (SOMNOlab für SOMNOcheck micro, Weinmann GmbH, Hamburg, Germany). Respiratory events were automatically detected and analysed as previously described [30]. AHI_Scm_ was calculated as the number of automatically scored apnoea and hypopnoea events divided by the recording time. Automatically scored apnoea was defined as follows: (1) ≥ 90% decrease in the flow amplitude compared to baseline; (2) duration of the event ≥ 10 s;  (3) > 90% of the event duration met the amplitude reduction criteria for apnoea. Automatically scored hypopnoea was defined as follows: (1) ≥ 50% decrease in the flow amplitude compared to baseline together with 3% oxygen desaturation or an autonomic arousal;  (2) duration of the event ≥ 10 s; (3) more than 90% of the event’s duration met the amplitude reduction criteria for hypopnoea [30]. Previous evaluation of the Somnocheck micro CARDIO^®^ system and a predecessor (Somnocheck system) in comparison to simultaneous PSG suggested sufficient diagnostic accuracy [24, 31, 32].

Patients were instructed by a medical-technical assistant trained according to the manufacturer’s protocol to use the SCm device. To minimize the confounding effects of an unfamiliar sleeping environment, polygraphic examination was performed overnight in conventional patient rooms on the psychiatric ward. The SCm device was applied with the assistance of a specialised psychiatric nurse immediately before the patient’s usual bedtime.

### Polysomnography

Polysomnographic measurements were performed by trained sleep lab technicians in the sleep laboratory of the Department of Internal Medicine 1, Friedrich-Alexander-Universität Erlangen-Nürnberg, Erlangen, Germany, as previously described [33]. Patients were monitored for at least 6 h. The measured parameters included electroencephalography, bilateral electrooculography, submental electromyography, electrocardiography, oxygen saturation measurement using a finger oximeter, nasal airflow measurement, microphone-based detection of snoring and detection of thoracic and abdominal movements. The following AHI cut-offs were adopted for PSG: any OSA (AHI ≥ 5), moderate to severe OSA (AHI ≥ 15), and severe OSA (AHI ≥ 30). The measured data were evaluated by a qualified sleep physician.

### Analysis

#### Data acquisition

Data on demographics, comorbidities, in-hospital parameters, and laboratory data were extracted from the patients’ medical records. To ensure the accuracy and consistency of the data, all medical records were cross-verified where discrepancies were noted, and standardized protocols were followed for data extraction. The data included age (in years), gender (male/female), Body Mass Index (BMI), smoking status (current/former/never), alcohol use (regular alcohol use defined as consumption more than three times a week), and medical comorbidities such as hypertension, diabetes, and cardiovascular disease. Psychiatric diagnoses were determined by the attending psychiatrist according to the ICD-10 criteria [34]. Specific diagnosis categories were defined as Affective Disorders (F30.0-2, F31.0-9, F32.0-9, F33.0-9, F34.0-1), Unipolar Depressive Disorder (F32.0-9, F33.0-9), Bipolar Disorder (F30.0-2, F31.0-9), Psychotic Disorders (F20.0-9, F22, F23.0-3, F24, F25.0-9), and Neurodegenerative Disorders (F00.0-2, F01.0-3, F02.0-4, F03, F06.7). The specific definitions and measurement methods for all variables, including demographic characteristics, clinical diagnoses, and measured parameters, are detailed in Supplementary Table 1. Data analysis and manuscript preparation were conducted in accordance with the Strengthening the Reporting of Observational Studies in Epidemiology (STROBE) guidelines for reporting observational studies.

### Outcomes

We evaluated the Apnoea-Hypopnoea-Index (mean number of apnoeas and hypopnoeas per hour of sleep (PSG) or measured time (SCm), AHI) measured by PSG and SCm. Other outcome parameters were the Apnoea-Index (mean number of apnoeas per hour of sleep (PSG) or measured time (SCm), AI), the Oxygen-Desaturation-Index (number of desaturation episodes (decrease in mean oxygen saturation of ≥ 3% over 120 s) per hour of sleep (PSG) or measured time (SCm), ODI) and the minimal oxygen saturation. We analysed clinical characteristics such as psychiatric and non-psychiatric diagnoses, psychiatric and non-psychiatric medications and established risk factors for OSA including adiposity, smoking and alcohol consumption [35]. Pre-existing comorbidities, including chronic obstructive pulmonary disease (COPD), asthma, pulmonary hypertension, arterial hypertension, atrial fibrillation, heart failure, kidney disorders, hypothyroidism, and gastroesophageal reflux disease, were documented due to their known associations with OSA and their relevance in potentially complicating its clinical presentation and treatment. [36, 37].

### Statistical analysis

Statistical analyses were performed using IBM SPSS Statistics, version 28.0 and Microsoft Excel, version 2019. Ordinal and continuous variables are presented as medians (IQRs), and categorical variables are presented as total numbers (percentages). Parameters were compared using the Wilcoxon signed-rank test for paired samples, with a two-tailed p-value less than 0.05 considered significant. Deviations from a normal distribution were assessed with the Shapiro-Wilk test. Due to the non-normal distribution of the analysed variables, Spearman correlation coefficients (r_s_) were calculated to assess correlations between the AHI, AI, ODI and minimum oxygen saturation measured by PSG and SCm. The sensitivity and specificity of the SCm device for the diagnosis of OSA were calculated by cross-tabulation (sensitivity = true positive / (true positive + false negative), specificity = true negative / (true negative + false positive). Diagnostic accuracy was assessed using the area under the receiver operating characteristic (ROC) curve. Optimal cut-off levels were determined using the Youden-Index [38]. Bland-Altman analysis was performed to assess the agreement between the AHI measured by SCm and PSG.

## Results

### Study population and baseline characteristics

**Fig. 1:**
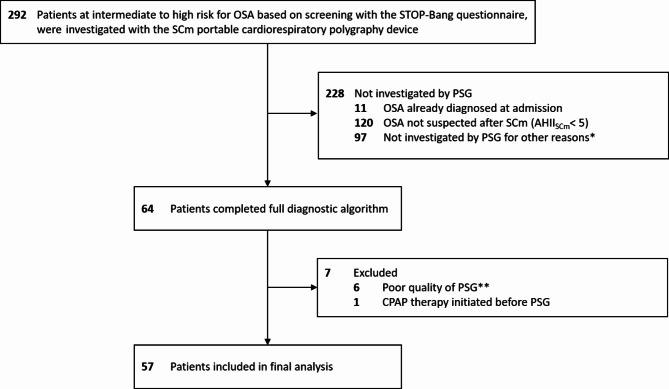
Flow chart of study participants. OSA indicates Obstructive Sleep Apnoea; SCm, Somnocheck micro CARDIO^®^ portable cardiorespiratory polygraphy device; PSG, polysomnography; AHI, Apnoea-Hypopnoea-Index; CPAP, Continuous Positive Airway Pressure. *. i.e. PSG was not available; patient status did not allow PSG measurement; patient did not consent to PSG; patient did not complete diagnostic algorithm for unknown reasons. **. Sleep duration < 200 min or poor quality was noted in medical records

A total of 292 patients were investigated with the SCm portable cardiorespiratory polygraphy device (Fig. 1). A total of 228 patients were not investigated by polysomnography (OSA was already diagnosed at admission, 11; OSA was not suspected after SCm measurement, 120; other reasons, 97). A total of 64 patients completed the full diagnostic algorithm including polysomnography, of whom 57 were included in the final analysis (median [IQR] age 62.0 [51.5–72.5] years; 23 [40.4%] women; 34 [59.6%] men). Baseline demographics and clinical characteristics are summarised in Table 1. The median STOP-Bang total score at baseline was 4.0 [IQR, 3–5]. Of the 57 patients included, 41 [71.9%] had an affective disorder as their main diagnosis. Antidepressants were used by 35 [61.4%], antipsychotics were used by 15 [26.3%] and sedatives were used by 13 [22.8%] patients. There was a significant prevalence of common risk factors for OSA: The median BMI was 27.4 kg/m² [IQR, 23.2–31.6]. Cardiovascular diseases were diagnosed in 34 [59.6%] patients. Antihypertensives were used in 33 [57.9%] patients. A total of 24 [42.1%] patients had a history of substance use (10 [17.5%] active nicotine users; 6 [10.5%] former smokers; 10 [17.5%] regular alcohol users). To ensure the representativeness of our results, we compared the baseline characteristics of the study cohort with those of the excluded samples and found no significant differences for the majority of variables, including STOP-Bang total sore, BMI and age at admission (Supplementary Table 2).

**Table 1:** Baseline demographics and clinical characteristics

Variable	Study population
Subjects	57
Demographics	
Sex	
Female, No. (%)	23 (40.4)
Male, No. (%)	34 (59.6)
Age, median (IQR), y	62.0 (51.5–72.5)
BMI, median (IQR), kg/m2	27.4 (23.2–31.6) *
Duration of Stay, median (IQR), d	42.0 (24.0–59.0)
Time from Admission to SCm, median (IQR), d	2.0 (1.0-5.5)
Time from Admission to PSG, median (IQR), d	13.0 (7.0–21.0)
Time between SCm and PSG, median (IQR), d	7.0 (4.0–17.0)
Private Insurance, No. (%)	53 (93.0)
BDI-II score at Admission, median (IQR)	29.0 (20.0–36.0) *
STOP-Bang total score, median (IQR)	4.0 (3.0–5.0)
Main Diagnosis	
Affective Disorder, No. (%)	41 (71.9)
Unipolar Depressive Disorder, No. (%)	38 (66.7)
Bipolar Disorder, No. (%)	3 (5.3)
Psychotic Disorders, No. (%)	3 (5.3)
Neurodegenerative Disorders, No. (%)	4 (7.0)
Psychiatric Medication	
Number of Medication at Admission, median (IQR)	1.0 (0-2.5)
Antidepressants, No. (%)	35 (61.4)
Antipsychotics, No. (%)	15 (26.3)
Lithium, No. (%)	3 (5.3)
Sedatives, No. (%) **	13 (22.8)
Anticonvulsants, No. (%)	5 (8.8)
Antidementia Drugs, No. (%)	2 (3.5)
Non-psychiatric Diseases	
Pulmonary Diseases, No. (%)	5 (8.8)
COPD, No. (%)	2 (3.5)
Asthma, No. (%)	2 (3.5)
Pulmonary Hypertension, No. (%)	1 (1.8)
Cardiovascular Diseases, No. (%)	34 (59.6)
Arterial Hypertension, No. (%)	31 (54.4)
Atrial Fibrillation, No. (%)	7 (12.3)
Heart Failure, No. (%)	3 (5.3)
Kidney Disorders, No. (%)	7 (12.3)
Hypothyroidism, No. (%)	16 (28.1)
Gastroesophageal Reflux Disease, No. (%)	2 (3.5)
Non-psychiatric Medication, No. (%)	42 (73.7)
Proton Pump Inhibitors, No. (%)	10 (17.5)
Antihypertensives, No. (%)	33 (57.9)
Beta-blockers, No. (%)	22 (38.6)
Substance Use History, No. (%) ***	24 (42.1)
Active Nicotine Use, No. (%) ^+^	10 (17.5)
Former Smoker, No. (%)^++^	6 (10.5)
Regular Alcohol Use, No. (%)^+++^	10 (17.5)

*. Missing values: BMI data were available for 54 of 57 patients; BDI-II scores were available for 46 of 57 patients

**. Sedatives were defined as patients taking zopiclone, zolpidem or benzodiazepines at admission

***. Substance Use History was defined as self-reported history of nicotine, alcohol, cannabinoid or illegal drug use at admission

^+^. Active Nicotine Use was defined as self-reported current active nicotine use or less than 6 months of abstinence of nicotine products

^++^. Former Smoker was defined as self-reported history of smoking, but current non-smoker for over 6 months

^+++^. Regular Alcohol Use was defined as self-reported regular consumption of alcohol, defined as consuming alcohol at least three times weekly

Ordinal and continuous variables are presented as medians (IQRs), categorical variables as total numbers (percentages). PSG indicates polysomnography; SCm, Somnocheck micro CARDIO^®^ portable cardiorespiratory polygraphy device; No., number; BMI, Body-Mass-Index; BDI-II, Beck Depression Inventory Version II; COPD, chronic obstructive pulmonary disease

### Sleep laboratory versus portable device testing

Regarding AHI, there were no significant differences between the non-simultaneous sleep laboratory and portable device measurements (AHI measured by PSG, median, 16.6 [IQR, 6.2–26.7] vs. AHI measured by SCm, median, 14.9 [IQR, 10.0-22.8]; *p* = 0.812) and a strong positive correlation (r_s_=0.71, *p* < 0,001) (Table 2 and Supplementary Fig. 1). There were significant differences in Apnoea-Index (AI), Oxygen-Desaturation-Index (ODI) and minimum oxygen saturation between the sleep laboratory and portable device measurements: The median AI was significantly higher in the SCM portable polygraphy (AI measured by PSG, median, 1.3 [IQR, 0-6.4] vs. AI measured by SCm, median, 6.9 [IQR, 3.6–14.5]; *p* < 0.001), with a modest positive correlation [r_s_=0.54, *p* < 0.001]. The median ODI was significantly lower in SCm portable polygraphy (ODI measured by PSG, median, 17.6 [IQR, 7.2–30.7] vs. ODI measured by SCm, median, 8.0 [IQR, 3.3–13.6]; *p* < 0.01), with a modest positive correlation [r_s_=0.62, *p* < 0.001]. Minimum peripheral oxygen saturation (SpO2) was slightly lower in SCm portable polygraphy (min. SpO2 measured by PSG, median, 83.0 [IQR, 79.5–86.0] vs. min. SpO2 measured by SCm, median, 81.0 [IQR, 77.0-84.5]; *p* = 0.012), with a weak positive correlation [r_s_=0.47, *p* < 0.001]. Bland-Altmann analysis demonstrated sufficient agreement between the AHI measured by sleep laboratory (AHI_PSG_) and the AHI measured by portable device (AHI_SCm_), with a mean difference of the AHI_PSG_ minus the AHI_SCm_ of 2.04 (Supplementary Fig. 2).

**Table 2:** Results of PSG and SCm Recording

	PSG	SCm	*p*	*r*_s_
Apnoea-Hypopnoea-Index (AHI), median (IQR)	16.6 (6.2–26.7)	14.9 (10.0-22.8)	0.812	0.71**
Apnoea-Index (AI), median (IQR)	1.3 (0-6.4)	6.9 (3.6–14.5)	< 0.001	0.54**
Oxygen-Desaturation-Index (ODI), median (IQR)	17.6 (7.2–30.7)	8.0 (3.3–13.6)	< 0.001	0.62**
min. SpO2, median (IQR)	83.0 (79.5–86.0)	81.0 (77.0-84.5)	0.012	0.47**

Variables are presented as medians (IQRs). PSG indicates polysomnography; SCm, Somnocheck micro CARDIO^®^ portable cardiorespiratory polygraphy device; SpO2, peripheral oxygen saturation. p-values were calculated using the Wilcoxon signed-rank test for paired samples. r-values are given for Spearman (r_s_). **. The correlation is significant at the 0.01 level (2-tailed). All variables differed significantly from normal distribution, as measured with the Shapiro-Wilk test.

### Diagnostic accuracy of the SCm portable polygraphy device for detecting OSA

**Fig. 2:**
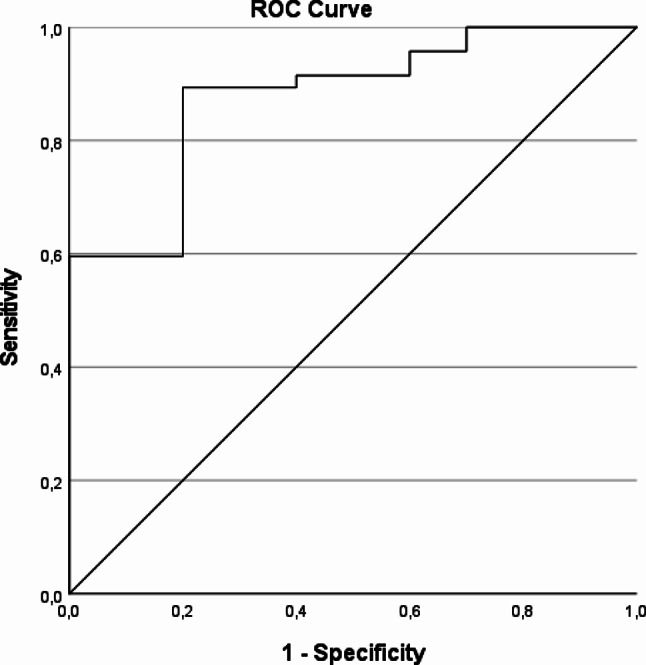
ROC curve analysis for the diagnosis of any OSA. Any OSA was defined as a measured AHI ≥ 5 in PSG. *n* = 57; AUC, 0.877; optimal threshold AHI_SCm_ ≥ 9.25; sensitivity, 0.894; specificity, 0.800; Youden-Index, J = 0.694. OSA indicates Obstructive Sleep Apnoea; AHI, Apnoea-Hypopnoea-Index; PSG, polysomnography; AUC, Area Under the Curve

Using standard cut-off values established for non-psychiatric patients (any OSA: AHI_SCm_ ≥ 5), SCm showed high sensitivity (0.957), but low specificity (0.300) for the diagnosis of any OSA (Table 3). ROC curve analysis was applied (Fig. 2; area under the curve (AUC), 0.877) to determine the optimal AHI_SCm_ cut-off value for the psychiatric study population (any OSA: AHI_SCm_ ≥ 9.25), after which SCm showed high sensitivity (0.894) and high specificity (0.800) for the diagnosis of any OSA (Table 3). To confirm the robustness of these findings, we conducted sensitivity analyses using additional OSA reference definitions. Specifically, ROC curve analysis was performed using the clinical rating by the examining sleep physician as a reference. The results were consistent with the main analysis, and these findings remained stable even after excluding patients whose AHI_PSG_ values did not align with the clinical ratings. (Suppl. Figure 3). Using the AHI_SCm_ cut-off values optimised for the psychiatric study population (Suppl. Figure 4), SCm showed acceptable sensitivity and specificity for the diagnosis of mild to moderate OSA (9.25 ≤ AHI_SCm_ < 22.75; sensitivity, 0.765; specificity, 0.826) and severe OSA (AHI_SCm_ ≥22.75; sensitivity, 0.769; specificity, 0.909) (Table 3).

**Table 3:** Diagnostic accuracy of the SCm portable polygraphy device for detecting OSA

	PSG (Reference)	SCm^1^			SCm_optimised_^2^		
	No. (%)	No. (%)	Sens.	Spec.	No. (%)	Sens.	Spec.
Any OSA	47 (82.5)	52 (91.2)	0.957	0.300	44 (77.2)	0.894	0.800
Mild to Moderate OSA	34 (59.6)	41 (71.9)	0.882	0.522	30 (52.6)	0.765	0.826
Severe OSA	13 (22.8)	11 (19.3)	0.692	0.955	14 (24.6)	0.769	0.909

^1^ SCm measurement using standard AHI_SCm_ cut-off values: Any OSA: AHI_SCm_ ≥ 5; Mild to Moderate OSA: 5 ≤ AHI_SCm_ < 30; Severe OSA: AHI_SCm_ ≥ 30

^2^ SCm measurement using optimised AHI_SCm_ cut-off values for the psychiatric study population: Any OSA: AHI_SCm_ ≥ 9.25; Mild to Moderate OSA: 9.25 ≤ AHI_SCm_ < 22.75; Severe OSA: AHI_SCm_ ≥ 22.75

Variables are presented as total numbers (percentages). PSG indicates polysomnography; SCm, Somnocheck micro CARDIO^®^ portable cardiorespiratory polygraphy device; No., number. Sensitivity (Sens.) and specificity (Spec.) were calculated for the diagnosis of OSA based on the AHI measured by the reference polysomnography using the following cut-off values: Any OSA: AHI_PSG_ ≥ 5; Mild to Moderate OSA: 5 ≤ AHI_PSG_ < 30; Severe OSA: AHI_PSG_ ≥ 30

## Discussion

This observational cohort study compared the Somnocheck micro CARDIO^®^ (SCm) portable polygraphy device to the gold standard in-laboratory polysomnography (PSG) to identify Obstructive Sleep Apnoea (OSA) among psychiatric patients in a real-world clinical setting. Our results indicated that the SCm device has a high diagnostic accuracy. A strong positive correlation was found between the SCm and PSG AHI measurements, and no significant differences were detected.

OSA is increasingly recognised for its impact on psychiatric patients. Psychiatric patients are at a high risk of OSA [5, 6]. Identifying OSA in psychiatric patients can be challenging due to symptom overlap with psychiatric disorders and common adherence problems [12–14]. Therefore, it is important to ensure that the diagnostic process is easily accessible for psychiatric patients.

Our study provides evidence that the SCm portable polygraphy device can effectively serve as both a screening method and an alternative to PSG in psychiatric patients at risk for OSA, particularly when in-laboratory diagnostic PSG may not be feasible. When standard cut-off values established for non-psychiatric patients (any OSA: AHI_SCm_ ≥ 5) were applied, the SCm device provided high sensitivity, but low specificity. Using the standard cut-offs, SCm can be used as a screening tool followed by confirmation of the diagnosis by in-laboratory PSG, applicable in both psychiatric and non-psychiatric settings. When optimised cut-off values determined for the psychiatric study population were applied, SCm demonstrated high diagnostic accuracy (sensitivity 89.4%, specificity 80.0%, AUC 0.877) to identify any OSA. With optimised cut-off values, the SCm measurement also allowed to evaluate OSA severity in psychiatric patients, thus identifying patients for whom treatment initiation is most critical.

While the diagnostic accuracy regarding the AHI was high, significant differences were observed between PSG and SCm in the Apnoea-Index, Oxygen-Desaturation-Index, and minimum oxygen saturation. The differences in the Apnoea- and Oxygen-Desaturation-Indices could be partially attributed to inter-night variability, given that parameters such as the ODI are known to vary significantly from night to night [39]. Accordingly, simultaneous recording by PSG and a predecessor of the SCm device showed stronger correlations for these parameters [31]. However, it is plausible that the automatic analysis in the SCm portable polygraphy is less accurate in differentiating between apnoeas and hypopnoeas than traditional PSG. Nevertheless, this does not appear to affect the overall diagnostic accuracy, as there were no significant differences regarding the AHI, and the differentiation between apnoeas and hypopnoeas has limited clinical significance [40, 41].

It seems likely that the diagnostic accuracy of the SCm device is underestimated in the current study due to the non-simultaneous recording of PSG and SCm in two different nights. Given the high inter-night variability in OSA severity parameters, such as the AHI [39, 42, 43], a simultaneous recording might have resulted in even stronger correlations. However, the real-world design of our study mirrors current clinical practice, where PSG is usually only conducted after an initial examination using a portable device, as recommend by current sleep medicine guidelines [26, 44]. Furthermore, the baseline characteristics of the study cohort and the excluded patients were comparable despite the sequential diagnostic algorithm, supporting the generalisability of the study´s findings. The high diagnostic accuracy regarding the AHI, despite the non-simultaneous recording, supports the use of portable polygraphy devices both as a screening method and as a diagnostic alternative to PSG in psychiatric patients.

The high specificity of the SCm portable polygraphy in patients with severe OSA (90.9%) suggests that these patients could potentially bypass diagnostic PSG and proceed directly to CPAP titration, thus streamlining the diagnostic and therapeutic process. This could be particularly beneficial in a psychiatric setting, where PSG is often impractical due to logistical and patient compliance issues. Furthermore, this approach could be extended to CPAP titration in an ambulatory or specialized psychiatric setting, as supported by evidence from a randomized controlled trial in the general population, which demonstrated non-inferiority, lower costs and shorter waiting periods for ambulatory CPAP titration [45]. Further studies should explore whether this approach could be applicable in a psychiatric setting.

While our findings clearly support the integration of polygraphic measurements into routine sleep assessments of psychiatric patients, this could also be extended to the use of consumer electronic devices. Wearables such as smartwatches are becoming increasingly popular and can provide valuable information for detecting OSA [46, 47]. However, further research is needed to validate the use of these devices against the conventional diagnostic algorithm using PSG [48, 49]. Given that wearables provide accurate measurements of pulse oximetry, snoring, and motion detection [50, 51], similar to the technologies used by the SCm device, the results of this study can be partially extrapolated to these devices. In the future, these devices could be used to identify additional patients at risk for OSA and monitor treatment in patients diagnosed with OSA. Other potential approaches include the use of smartphone apps as screening tools [52]. A potential optimised diagnostic algorithm could include an initial screening using questionnaires and data extracted from the patients consumer electronic device, followed by diagnostic polygraphy in patients at elevated risk for OSA. This could make the diagnostic procedure more accessible and cost-efficient, which would be particularly useful in a psychiatric setting.

This study has several limitations. The relatively small sample size may limit the power to detect statistically significant differences. Additionally, the non-simultaneous recording of PSG and SCm measurements on different nights introduces variability due to known inter-night differences in OSA severity. Given that polysomnography was conducted following the initial screening with the STOP-Bang questionnaire and the SCm device, the pre-test probability of detecting OSA was relatively high. Other limitations include the extended data collection period and the observational study design. Despite these limitations, the study provides valuable real-world data and underscores the practical applications of portable polygraphy devices in clinical settings. Future research with larger, more diverse samples and simultaneous recording methodologies is needed to confirm these findings and further evaluate the diagnostic accuracy of portable polygraphy devices.

## Conclusions

This study demonstrated that portable polygraphy devices such as the Somnocheck micro CARDIO^®^ (SCm) can provide an accurate diagnostic alternative to the gold standard polysomnography (PSG) for identifying obstructive sleep apnoea (OSA) in psychiatric patients. Given the high prevalence of OSA among psychiatric patients and the challenges in diagnosing it due to symptom overlap and adherence issues, the availability of an accessible diagnostic procedure such as the SCm device is particularly important. Our findings suggest that in a psychiatric setting, the SCm device can effectively serve as both a screening method and an alternative to diagnostic PSG. This study supports the integration of portable polygraphic measurements into routine sleep assessments for psychiatric patients, which could significantly improve the identification and management of OSA in this population. Additionally, extending these findings to consumer electronic devices, such as wearables, may further increase the accessibility and cost-efficiency of OSA diagnosis and monitoring. Ultimately, early and accurate detection of OSA among psychiatric patients can greatly enhance the management of both their sleep disorders and mental health issues, potentially leading to better treatment outcomes and improved quality of life.

## Electronic supplementary material

Below is the link to the electronic supplementary material.

Supplementary Material 1

Supplementary Material 2

## Data Availability

The datasets generated during and/or analysed during the current study are available from the corresponding author upon reasonable request.

## Abbreviations

AHI: Apnoea-Hypopnoea-Index
AI: Apnoea-Index
AUC: Area Under the Curve
BDI-II: Beck Depression Inventory Version II
BMI: Body-Mass-Index
COPD: Chronic Obstructive Pulmonary Disease
CPAP: Continuous Positive Airway Pressure
IQR: Interquartile Range
ODI: Oxygen-Desaturation-Index
OSA: Obstructive Sleep Apnoea
PSG: Polysomnography
ROC: Receiver Operating Characteristic
SCm: Somnocheck micro CARDIO^®^ portable cardiorespiratory polygraphy device
Sens.: Sensitivity
Spec.: Specificity
SpO2: Peripheral Oxygen Saturation
STROBE: Strengthening the Reporting of Observational Studies in Epidemiology
UKER-OSA-PS: Universitätsklinikum Erlangen study of obstructive sleep apnoea in psychiatric patients
