# Supplementary material for: Portable polygraphic device (Somnocheck micro CARDIO®) provides accurate diagnostic information in psychiatric patients at risk for obstructive sleep apnoea: an observational cohort study
Source: BMC Psychiatry. 2024 Sep 10;24:607. doi: 10.1186/s12888-024-06049-8 (PMC11389046; doi:10.1186/s12888-024-06049-8)
Supplement: Supplementary file 1 — Supplementary Material 1 [file 12888_2024_6049_MOESM1_ESM.docx]

**Supplementary Figures**

**Supplementary Figure 1. Scatterplot of Apnoea-Hypopnoea-Index measured by SCm and PSG.**


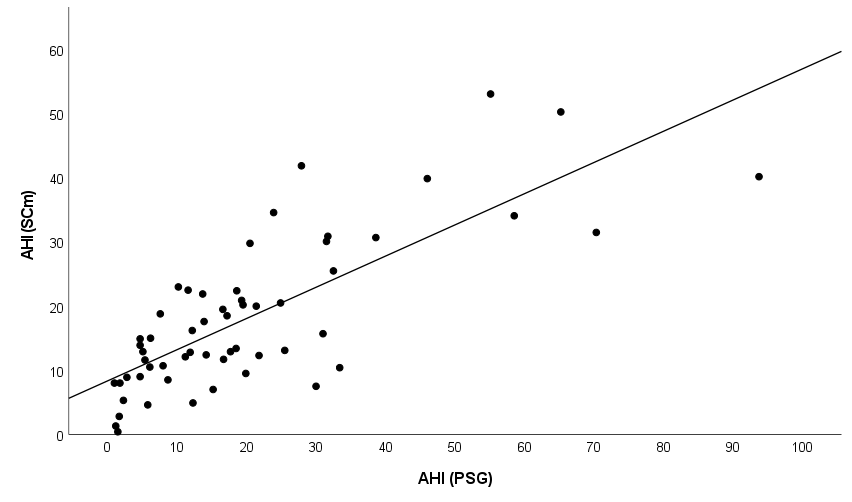


AHI indicates Apnoea-Hypopnoea-Index; PSG, polysomnography; SCm, Somnocheck micro CARDIO® portable cardiorespiratory polygraphy device.

**Supplementary Figure 2: Bland-Altman Plot comparing Apnoea-Hypopnoea-Index (AHI) measurements between Somnocheck micro CARDIO® (SCm) and Polysomnography (PSG)**.


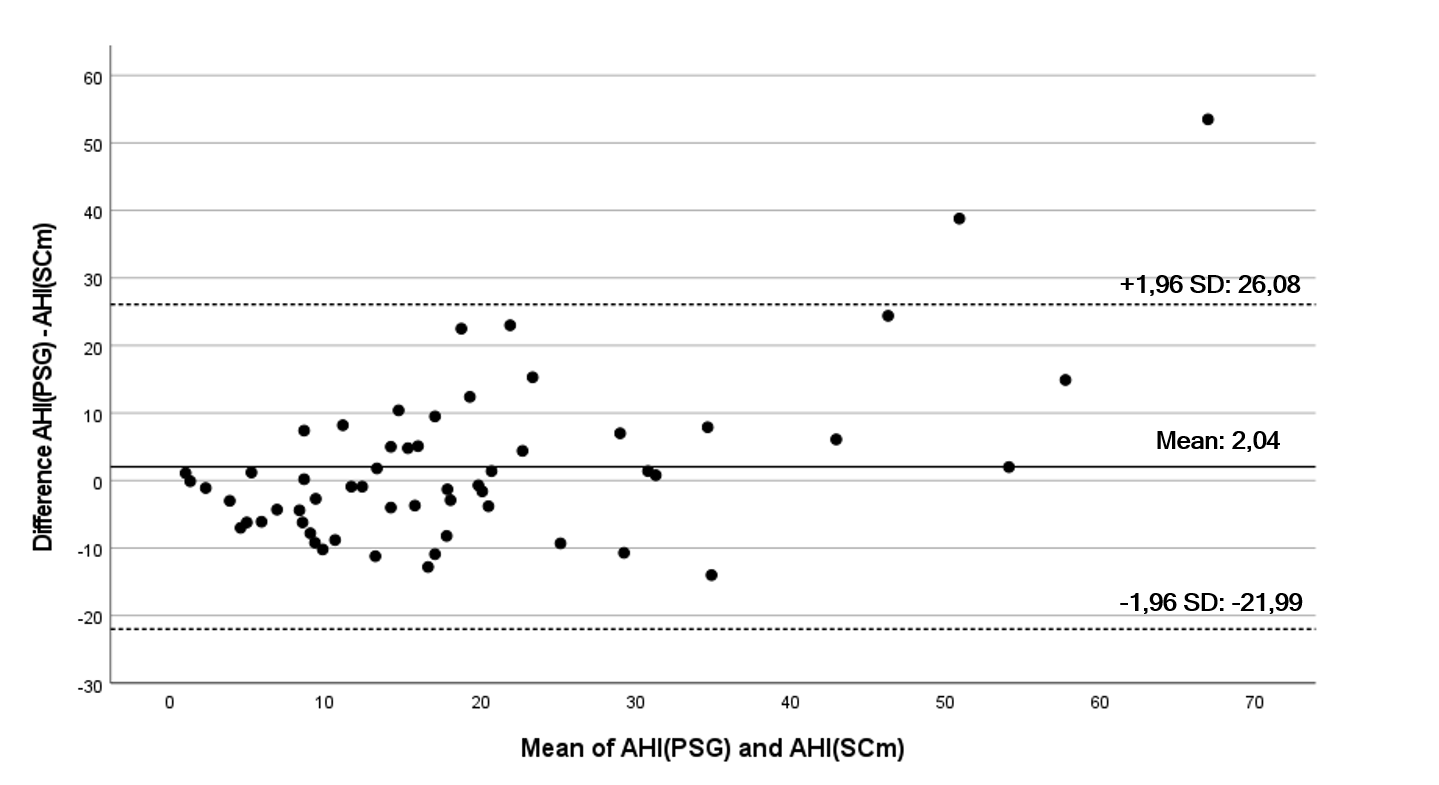


AHI indicates Apnoea-Hypopnoea-Index; PSG, polysomnography; SCm, Somnocheck micro CARDIO® portable cardiorespiratory polygraphy device. The Bland-Altman plot was constructed by plotting the differences between SCm and PSG AHI values against their mean. Y-axis, difference in AHI values (AHI_PSG_ - AHI_SCm_); X-axis, mean AHI values obtained from both methods; solid line, mean difference, 2.04; dashed lines, limits of agreement (mean difference ± 1.96 standard deviations, 26.08 and -21.99).

**Supplementary Figure 3. Sensitivity analyses for additional OSA reference definitions**


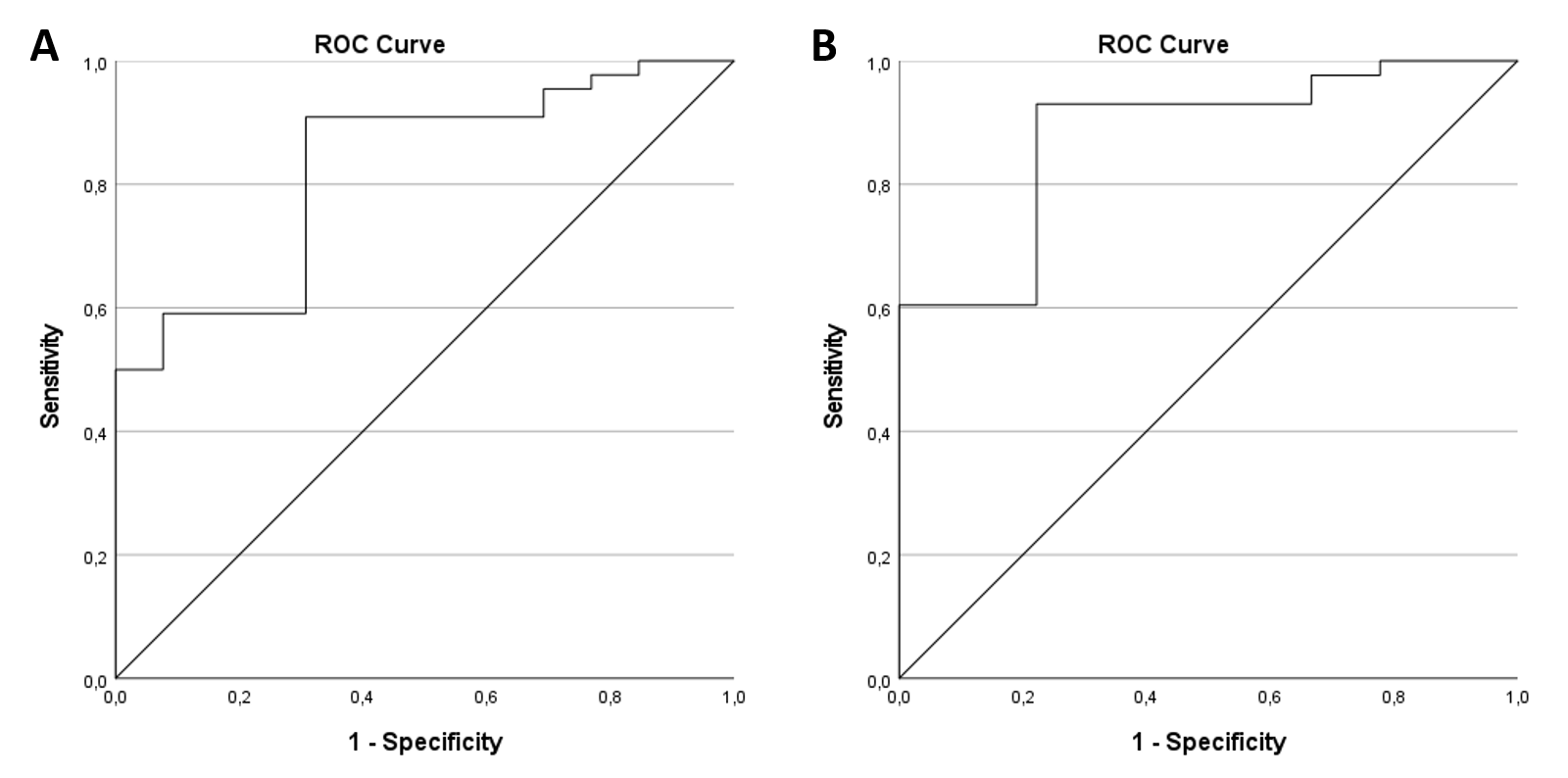


**A:** ROC curve analysis for the diagnosis of OSA based on the clinical rating by the examining sleep physician, including visual analysis of flow, effort and oxygen saturation and evaluation of the severity of clinical symptoms such as daytime sleepiness. n=57; AUC, 0.827; optimal lower threshold ≥ 9.25; sensitivity, 0.909; specificity, 0.692; Youden-Index, J=0.601.

**B:** ROC curve analysis for the diagnosis of OSA based on a measured AHI >5 in PSG, excluding patients with incongruent AHI_PSG_ and clinical assessment results (diagnosed with OSA by the examining sleep physician despite an AHI_PSG_ of less than 5 or not diagnosed with OSA despite having an AHI_PSG_ greater than 5). n=52; AUC, 0.879; optimal lower threshold ≥ 9.25; sensitivity, 0.930; specificity 0.778; Youden-Index, J=0.708.

**Supplementary Figure 4.** **ROC curve analysis for the diagnosis of moderate to severe OSA.**


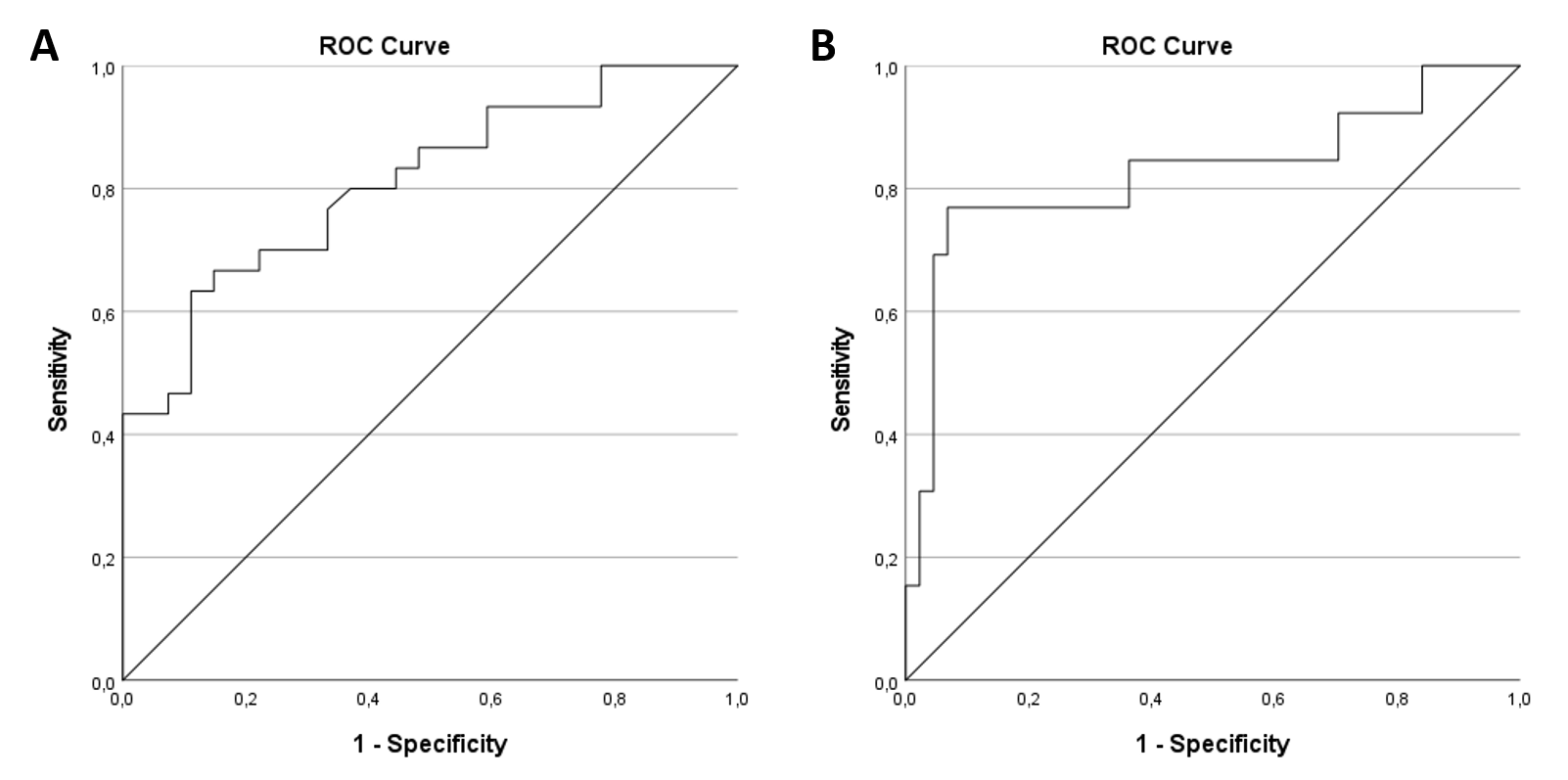


**A:** Diagnosis of moderate to severe OSA was defined as a measured AHI ≥ 15 in PSG. n=57; AUC, 0.810; optimal threshold ≥ 19.15; sensitivity, 0.633; specificity, 0.889; Youden-Index, J=0.522.

**B:** Diagnosis of severe OSA was defined as a measured AHI ≥ 30 in PSG. n=57; AUC, 0.827; optimal threshold ≥ 22.75; sensitivity, 0.769; specificity 0.909; Youden-Index, J=0.678.
