# Supplementary material for: Portable polygraphic device (Somnocheck micro CARDIO®) provides accurate diagnostic information in psychiatric patients at risk for obstructive sleep apnoea: an observational cohort study
Source: BMC Psychiatry. 2024 Sep 10;24:607. doi: 10.1186/s12888-024-06049-8 (PMC11389046; doi:10.1186/s12888-024-06049-8)
Supplement: Supplementary file 2 — Supplementary Material 2 [file 12888_2024_6049_MOESM2_ESM.docx]

**Supplementary Table 1. Definitions and measurement methods for variables**

| **Variable** | **Definition** | **Measurement Method** |
| --- | --- | --- |
| **Demographics** |  |  |
| Gender | Patient's identified gender, recorded as Male or Female | Recorded from medical records |
| Age | Number of years at the time of study enrolment | Recorded from medical records |
| Body Mass Index (BMI) | Weight in kilograms divided by height in meters squared (kg/m²) at admission | Calculated from measured or self-reported height and weight |
| Duration of Stay | Total duration of inpatient stay at study centre, reported in days | Recorded from medical records |
| Time from Admission to SCm | Time from hospital admission to SCm testing, reported in days | Recorded from medical records |
| Time from Admission to PSG | Time from hospital admission to PSG testing, reported in days | Recorded from medical records |
| Time between SCm and PSG | Interval between SCm and PSG testing, reported in days | Recorded from medical records |
| Private Insurance | Status of private insurance coverage | Recorded from medical records, reported as number and percentage |
| BDI-II score at Admission | Beck Depression Inventory-II score | Patient self-report, reported as median with interquartile range (IQR) |
| **Main Psychiatric Diagnosis** | Main diagnosis according to the ICD-10 criteria |  |
| Affective Disorder | Diagnosis of an Affective Disorder according to the ICD-10 criteria (F30.0-2, F31.0-9, F32.0-9, F33.0-9, F34.0-1) | Recorded from medical records, reported as number and percentage |
| Unipolar Depressive Disorder | Diagnosis of Unipolar Depressive Disorder according to the ICD-10 criteria ( F32.0-9, F33.0-9) | Recorded from medical records, reported as number and percentage |
| Bipolar Disorder | Diagnosis of Bipolar Disorder according to the ICD-10 criteria (F30.0-2, F31.0-9) | Recorded from medical records, reported as number and percentage |
| Psychotic Disorders | Diagnosis of a Psychotic Disorder according to the ICD-10 criteria (F20.0-9, F22, F23.0-3, F24, F25.0-9) | Recorded from medical records, reported as number and percentage |
| Neurodegenerative Disorders | Diagnosis of a Neurodegenerative Disorder according to the ICD-10 criteria (F00.0-2, F01.0-3, F02.0-4, F03, F06.7) | Recorded from medical records, reported as number and percentage |
| **Medication at admission** | Use of psychiatric medication at admission |  |
| Antidepressants | Use of antidepressant medications, including SSRIs, SNRIs, etc. | Recorded from medical records, reported as number and percentage |
| Antipsychotics | Use of antipsychotic medications | Recorded from medical records, reported as number and percentage |
| Lithium | Use of lithium | Recorded from medical records, reported as number and percentage |
| Sedatives | Use of sedatives was defined as patients taking zopiclone, zolpidem or benzodiazepines. | Recorded from medical records, reported as number and percentage |
| Anticonvulsants | Use of anticonvulsant medications | Recorded from medical records, reported as number and percentage |
| Antidementia drugs | Use of drugs for dementia, such as cholinesterase inhibitors | Recorded from medical records, reported as number and percentage |
| **Non-psychiatric Diseases** | Self-reported or clinically diagnosed non-psychiatric comorbidities at admission according to ICD-10 criteria |  |
| Pulmonary Diseases | Diagnosis of any pulmonary diseases | Recorded from medical records, reported as number and percentage |
| COPD | Diagnosis of chronic obstructive pulmonary disease | Recorded from medical records, reported as number and percentage |
| Asthma | Diagnosis of asthma | Recorded from medical records, reported as number and percentage |
| Pulmonary Hypertension | Diagnosis of pulmonary hypertension | Recorded from medical records, reported as number and percentage |
| Cardiovascular Diseases | Presence of any cardiovascular diseases | Recorded from medical records, reported as number and percentage |
| Arterial Hypertension | Diagnosis of arterial hypertension | Recorded from medical records, reported as number and percentage |
| Atrial Fibrillation | Diagnosis of atrial fibrillation | Recorded from medical records, reported as number and percentage |
| Heart Failure | Diagnosis of heart failure | Recorded from medical records, reported as number and percentage |
| Kidney Disorders | Presence of any kidney disorders | Recorded from medical records, reported as number and percentage |
| Hypothyroidism | Diagnosis hypothyroidism | Recorded from medical records, reported as number and percentage |
| Gastroesophageal Reflux Disease | Diagnosis of gastroesophageal reflux disease | Recorded from medical records, reported as number and percentage |
| **Non-psychiatric Medication** | Use of medication for non-psychiatric conditions at admission | Recorded from medical records, reported as number and percentage |
| Proton Pump Inhibitors | Use of proton pump inhibitors | Recorded from medical records, reported as number and percentage |
| Antihypertensives | Use of any antihypertensives | Recorded from medical records, reported as number and percentage |
| Beta-blockers | Use of beta-blocker medications | Recorded from medical records, reported as number and percentage |
| **Other Variables** |  |  |
| Substance Use History | Self-reported history of nicotine, alcohol, cannabinoid or illegal drug use at admission | Recorded from medical records, reported as number and percentage |
| Active Nicotine Use | Self-reported current active nicotine use or less than 6 months of abstinence of nicotine products | Recorded from medical records, reported as number and percentage |
| Former Smoker | Self-reported history of smoking, but current non-smoker for over 6 months | Recorded from medical records, reported as number and percentage |
| Regular Alcohol Use | Self-reported regular consumption of alcohol, defined as consuming alcohol at least three times weekly | Recorded from medical records, reported as number and percentage |
| **OSA Variables** |  |  |
| Apnoea-Hypopnoea Index (AHI) | Index (mean number of apnoeas and hypopnoeas per hour of sleep (PSG) or measured time (SCm) | Measured by PSG/SCm |
| Apnoea Index (AI) | Mean number of apnoeas per hour of sleep (PSG) or measured time (SCm) | Measured by PSG/SCm |
| Oxygen Desaturation Index (ODI) | Number of desaturation episodes, defined as ≥ 3% decrease in SpO2 for at least 10 seconds, per hour of sleep (PSG) or measured time (SCm) | Measured by PSG/SCm |
| min. SpO2 | Minimum oxygen saturation, recorded as the lowest oxygen saturation level during the measurement, in % | Measured by PSG/SCm |

**Supplementary Table 2. Comparison of Baseline Demographics and Clinical Characteristics between Study Population and Excluded Samples.**

| **Variable** | **Study Population** | **Excluded Patients** | **p** |
| --- | --- | --- | --- |
|  |  |  |  |
| Subjects | 57 | 235 |  |
| Demographics |  |  |  |
| Sex |  |  |  |
| Female, No. (%) | 23 (40.4) | 98 (41.7) | 0.85 |
| Male, No. (%) | 34 (59.6) | 137 (58.3) | 0.85 |
| Age, median (IQR), y | 62.0 (51.5-72.5) | 63.0 (52.0-73.0) | 0.69 |
| BMI, median (IQR), kg/m2 | 27.4 (23.2-31.6) * | 26.1 (23.4-29.6)* | 0.11 |
| Duration of Stay, median (IQR), d | 42.0 (24.0-59.0) | 23.0 (2.0-43.0) | **< 0.001** |
| Private Insurance, No. (%) | 53 (93.0) | 195 (83.0) | 0.06 |
| BDI-II score at Admission, median (IQR) | 29.0 (20.0-36.0) * | 22.0 (10.0-33.0)* | **0.02** |
| STOP-Bang total score, median (IQR) | 4.0 (3.0-5.0) | 4.0 (3.0-4.0)* | 0.07 |
| Main Diagnosis |  |  |  |
| Affective Disorder, No. (%) | 41 (71.9) | 134 (57.0) | **0.04** |
| Unipolar Depressive Disorder, No. (%) | 38 (66.7) | 120 (51.1) | **0.03** |
| Bipolar Disorder, No. (%) | 3 (5.3) | 14 (6.0) | 0.84 |
| Psychotic Disorders, No. (%) | 3 (5.3) | 9 (3.8) | 0.62 |
| Neurodegenerative Disorders, No. (%) | 4 (7.0) | 77 (32.8) | **< 0.001** |
| Psychiatric Medication |  |  |  |
| Number of Medication at Admission, median (IQR) | 1.0 (0-2.5) | 1.0 (0-2.0) | 0.33 |
| Antidepressants, No. (%) | 35 (61.4) | 122 (51.9) | 0.20 |
| Antipsychotics, No. (%) | 15 (26.3) | 57 (24.3) | 0.75 |
| Lithium, No. (%) | 3 (5.3) | 8 (3.4) | 0.70 |
| Sedatives, No. (%) ** | 13 (22.8) | 49 (20.9) | 0.75 |
| Anticonvulsants, No. (%) | 5 (8.8) | 24 (10.2) | 0.74 |
| Antidementia drugs, No. (%) | 2 (3.5) | 8 (3.4) | 0.97 |
| Non-psychiatric Diseases |  |  |  |
| Pulmonary Diseases, No. (%) | 5 (8.8) | 26 (11.1) | 0.61 |
| COPD, No. (%) | 2 (3.5) | 5 (2.1) | 0.63 |
| Asthma, No. (%) | 2 (3.5) | 14 (6.0) | 0.55 |
| Pulmonary Hypertension, No. (%) | 1 (1.8) | 1 (0.4) | 0.35 |
| Cardiovascular Diseases, No. (%) | 34 (59.6) | 122 (51.9) | 0.29 |
| Arterial Hypertension, No. (%) | 31 (54.4) | 112 (47.7) | 0.36 |
| Atrial Fibrillation, No. (%) | 7 (12.3) | 13 (5.5) | 0.07 |
| Heart Failure, No. (%) | 3 (5.3) | 5 (2.1) | 0.36 |
| Kidney Disorders, No. (%) | 7 (12.3) | 15 (6.4) | 0.16 |
| Hypothyroidism, No. (%) | 16 (28.1) | 49 (20.9) | 0.24 |
| Gastroesophageal Reflux Disease, No. (%) | 2 (3.5) | 3 (1.3) | 0.24 |
| Non-psychiatric Medication, No. (%) | 42 (73.7) | 177 (75.3) | 0.80 |
| Proton Pump Inhibitors, No. (%) | 10 (17.5) | 28 (11.9) | 0.26 |
| Antihypertensives, No. (%) | 33 (57.9) | 106 (45.1) | 0.08 |
| Beta-blockers, No. (%) | 22 (38.6) | 54 (23.0) | **0.02** |
| Substance Use History, No. (%) *** | 24 (42.1) | 114 (48.5) | 0.39 |
| Active Nicotine Use, No. (%) ^+^ | 10 (17.5) | 57 (24.3) | 0.28 |
| Former Smoker, No. (%)^++^ | 6 (10.5) | 35 (14.9) | 0.40 |
| Regular Alcohol Use, No. (%)^+++^ | 10 (17.5) | 41 (17.4) | 0.99 |

*. Missing values: BMI data were available for 54 of 57 patients (Study Population) and 132 of 235 patients (Excluded Patients); BDI-II scores were available for 46 of 57 patients (Study Population) and 184 of 235 patients (Excluded Patients); STOP-Bang total scores were available for 234 of 235 patients (Excluded Patients) .

**. Sedatives were defined as patients taking zopiclone, zolpidem or benzodiazepines at admission.

***. Substance Use History was defined as self-reported history of nicotine, alcohol, cannabinoid or illegal drug use at admission.

^+^. Active Nicotine Use was defined as self-reported current active nicotine use or less than 6 months of abstinence of nicotine products.

^++^. Former Smoker was defined as self-reported history of smoking, but current non-smoker for over 6 months.

^+++^. Regular Alcohol Use was defined as self-reported regular consumption of alcohol, defined as consuming alcohol at least three times weekly.

Ordinal and continuous variables are presented as medians (IQRs), categorical variables as total numbers (percentages). p-values were calculated using the Mann-Whitney U test for ordinal and continuous variables, while p-values for categorial variables were calculated using the Pearson Chi-Square test or Fisher`s Exact test (when the expected count was less than 5). PSG indicates polysomnography; SCm, Somnocheck micro CARDIO® portable cardiorespiratory polygraphy device; No., number; BMI, Body-Mass-Index; BDI-II, Beck Depression Inventory Version II; COPD, chronic obstructive pulmonary disease.

To ensure the representativeness of our results, we compared the baseline characteristics of the study cohort with those of the excluded samples. No significant differences were found for the majority of variables. For instance, age (study population, median, 62.0 [IQR, 51.5-72.5] vs. excluded patients, median, 63.0 [IQR, 52.0-73.0]; p=0.69), and BMI (study population, median, 27.4 [IQR, 23.2-31.6] vs. excluded patients, median, 26.1 [IQR, 23.4-29.6]; p=0.11) did not differ significantly. Most importantly, the STOP-Bang scores were comparable between the groups (study population, median, 4.0 [IQR, 3.0-5.0] vs. excluded patients, median, 4.0 [IQR, 3.0-4.0]; p=0.07), indicating that our study cohort is representative despite the sequential diagnostic algorithm.

However, some significant differences were noted. There was a larger proportion of patients with neurodegenerative disorders among the excluded patients (study population, 4 [7.0%] vs. excluded patients, 77 [32.8%]; p<0.001), and a smaller proportion of patients with affective disorders (study population, 41 [71.9%] vs. excluded patients, 134 [57.0%]; p=0.04). This can be explained by the fact that at the study site, patients with neurodegenerative disorders are usually admitted for only 2 days to perform a diagnostic spinal tap and are dismissed afterward, making it impractical to perform overnight PSG in the sleep laboratory. Consequently, the duration of stay was significantly shorter for excluded patients (study population, median, 42.0 [IQR, 24.0-59.0] vs. excluded patients, median, 23.0 [IQR, 2.0-43.0]; p<0.001) and the BDI-II scores were lower (study population, median, 29.0 [IQR, 20.0-36.0] vs. excluded patients, median, 22.0 [IQR, 10.0-33.0]; p=0.02). The only other notable difference was a higher use of beta-blockers among the included patients (study population, 22 [38.6%] vs. excluded patients, 54 [23.0%]; p=0.02), for which we currently have no specific explanation.

These findings suggest that while there were some differences between the included and excluded patients, the essential characteristics related to OSA risk assessment were comparable. This supports the representativeness of our study cohort despite the sequential diagnostic algorithm.
